# Which diagnostic tests are most useful in a chest pain unit protocol?

**DOI:** 10.1186/1471-227X-5-6

**Published:** 2005-08-25

**Authors:** Steve Goodacre, Thomas Locker, Jane Arnold, Karen Angelini, Francis Morris

**Affiliations:** 1 Medical Care Research Unit, Regent Court, 30 Regent Street, Sheffield, S1 4DA, UK; 2 Emergency Department, Northern General Hospital, Herries Road, Sheffield, S5 7AU, UK

## Abstract

**Background:**

The chest pain unit (CPU) provides rapid diagnostic assessment for patients with acute, undifferentiated chest pain, using a combination of electrocardiographic (ECG) recording, biochemical markers and provocative cardiac testing. We aimed to identify which elements of a CPU protocol were most diagnostically and prognostically useful.

**Methods:**

The Northern General Hospital CPU uses 2–6 hours of serial ECG / ST segment monitoring, CK-MB(mass) on arrival and at least two hours later, troponin T at least six hours after worst pain and exercise treadmill testing. Data were prospectively collected over an eighteen-month period from patients managed on the CPU. Patients discharged after CPU assessment were invited to attend a follow-up appointment 72 hours later for ECG and troponin T measurement. Hospital records of all patients were reviewed to identify adverse cardiac events over the subsequent six months. Diagnostic accuracy of each test was estimated by calculating sensitivity and specificity for: 1) acute coronary syndrome (ACS) with clinical myocardial infarction and 2) ACS with myocyte necrosis. Prognostic value was estimated by calculating the relative risk of an adverse cardiac event following a positive result.

**Results:**

Of the 706 patients, 30 (4.2%) were diagnosed as ACS with myocardial infarction, 30 (4.2%) as ACS with myocyte necrosis, and 32 (4.5%) suffered an adverse cardiac event. Sensitivities for ACS with myocardial infarction and myocyte necrosis respectively were: serial ECG / ST segment monitoring 33% and 23%; CK-MB(mass) 96% and 63%; troponin T (using 0.03 ng/ml threshold) 96% and 90%. The only test that added useful prognostic information was exercise treadmill testing (relative risk 6 for cardiac death, non-fatal myocardial infarction or arrhythmia over six months).

**Conclusion:**

Serial ECG / ST monitoring, as used in our protocol, adds little diagnostic or prognostic value in patients with a normal or non-diagnostic initial ECG. CK-MB(mass) can rule out ACS with clinical myocardial infarction but not myocyte necrosis(defined as a troponin elevation without myocardial infarction). Using a low threshold for positivity for troponin T improves sensitivity of this test for myocardial infarction and myocardial necrosis. Exercise treadmill testing predicts subsequent adverse cardiac events.

## Background

The chest pain unit (CPU) has been developed to provide standardised care for patients presenting with acute chest pain, undiagnosed by initial clinical assessment, electrocardiogram (ECG) and chest radiograph. The CPU aims to rapidly diagnose acute coronary syndrome (ACS), providing early access to appropriate care for those with positive test results and discharge home for those who test negative. Although a variety of different tests have been used in CPU protocols, most units use a combination of ECG monitoring, biochemical diagnostic testing and provocative cardiac testing to diagnose ACS [1]. This has led to some debate regarding the most appropriate testing regime. In particular, the role of the exercise treadmill test has been questioned [2] and does not feature in some protocols [3].

Since 1999 a CPU has been operating at the Northern General Hospital in Sheffield. Patients are investigated with a combination of serial ECG recording, ST segment monitoring, biochemical cardiac markers (CK-MB(mass) and troponin T) and an exercise treadmill test [4]. We aimed to determine the value of each of the constituent elements of this protocol by measuring: a) the diagnostic accuracy (sensitivity, specificity and likelihood ratios) of each test for ACS at presentation, and b) prognostic value of each test for major adverse cardiac events over six months.

## Methods

The Northern General Hospital is a 1100-bedded urban teaching hospital with the only adult emergency department for the 530,000 population of Sheffield, United Kingdom. The emergency department see approximately 90,000 new patients per year, of whom approximately 6% have chest pain. The hospital is a tertiary referral centre for cardiology, with cardiac catheterisation and cardiac surgery facilities. The CPU is a two-bedded, nurse-run unit based in the emergency department. Patients are assessed on the unit if they present with acute chest pain and have a normal or non-diagnostic ECG, no co-morbidity requiring admission, no serious alternative cause for chest pain (such as pulmonary embolus) and pain that is potentially compatible with cardiac ischaemia (i.e. not chest wall pain). Patients with known coronary heart disease can be assessed on the CPU provided they do not have prolonged (> one hour) or recurrent (more than one episode) pain that is characteristic of their angina.

The diagnostic testing regime consists of ST segment monitoring and serial ECG recording every hour for two to six hours; CK-MB(mass) assay on arrival, repeated either two hours later or six hours after symptom onset, whichever is later; troponin T assay at least six hours after symptom onset; and, if appropriate, exercise treadmill testing. Exercise testing is not performed if any preceding test is positive, if the patient has had recent diagnostic testing for coronary heart disease (e.g. coronary angiography), or if they are unable to exercise.

From 1/3/99 to 30/9/00 presenting details, CPU processes and diagnostic test results were prospectively recorded for all patients assessed on the CPU. Patients admitted after assessment received further testing and management at the discretion of the admitting physician and were followed up by case note review. Patients discharged after assessment were invited to attend a review appointment on the CPU 72 hours later for clinical assessment, ECG and troponin T measurement.

Six months after assessment the emergency department computer database was searched for details of any further hospital attendances or admissions. Case notes were reviewed for all cases identified. The general practitioners of all patients presenting between 1/3/99 and 29/2/00 were contacted by post to determine if they had suffered any adverse cardiac event or received diagnostic cardiac testing or procedures during the previous six months. This survey revealed no previously unidentified episodes so for patients attending from 1/3/00 to 30/9/00 the general practitioner was only contacted if the patient resided outside the Sheffield area. We did not attempt to contact patients at six months to confirm whether they were still alive.

ST segment monitoring and serial ECG recording was performed using Spacelabs monitors. These functions will be evaluated together, because: 1) serial ECG recording is facilitated and influenced by the availability of this monitoring equipment, and 2) ECG changes detected on ST segment monitoring may also be detected on serial recording and vice-versa. Each patient received between two and six hours of ST segment monitoring and at least two ECGs, one hour apart. Results were dichotomised into positive (>1 mm ST segment elevation or depression; T wave inversion > 2 mm or normalisation; ventricular arrhythmia; or second or third degree heart block) or negative (none of these changes). ECGs were interpreted by the specialist chest pain nurses who managed the unit. Positive changes were defined a priori and used for CPU decision-making.

The absolute value of CK-MB(mass) at baseline and on repeat sampling was defined as positive if it exceeded 5 ng/ml. The rise in CK-MB(mass) (delta CK-MB(mass)) was defined as positive if it exceeded 1.6 ng/ml. The diagnostic performance of baseline, repeat assay and change in CK-MB(mass) were analysed separately and then combined to give an overall result. This was defined as positive if either assay or the change were positive, and negative if all three results were negative. Two thresholds for positivity were analysed for troponin T: 1) the traditional threshold of 0.1 ng/ml or above, and 2) a more sensitive threshold of 0.03 ng/l. The CK-MB(mass) thresholds and the 0.1 ng/ml threshold for troponin were defined a priori and used for decision making on the CPU. The 0.03 ng/ml troponin threshold was only used for post hoc analysis.

The exercise treadmill test was performed according to the Bruce protocol. A positive test was defined as more than 1 mm horizontal or down-sloping ST segment depression; more than 1 mm ST elevation; or ventricular arrhythmia. For the purposes of this analysis, any test that did not produce these changes was considered to be negative, regardless of the duration of exercise or heart rate achieved.

The diagnostic performance of all tests except the exercise treadmill test was evaluated by comparison to the reference standard diagnosis of ACS at presentation, categorised as recently recommended [5] into: 1) ACS with clinical myocardial infarction (defined as a typical clinical syndrome associated with a troponin T elevation greater than 1.0 ng/ml), or 2) ACS with myocyte necrosis (defined as a typical clinical syndrome with any detectable troponin T less than 1.0 ng/ml). Sensitivity, specificity, positive and negative predictive values, and likelihood ratios were calculated for each test, along with 95% confidence intervals.

The prognostic value of each test was evaluated by measuring the relative risk of a major adverse cardiac event during the six months following a positive result, compared to a negative result. The following were defined as major adverse cardiac events: cardiac death, non-fatal myocardial infarction, arrhythmia, or revascularisation procedure. Because revascularisation procedures may be precipitated by the diagnostic tests under investigation, we repeated the analysis with these excluded from the definition.

Data were analysed using SPSS for windows version 11.5 (SPSS Inc., Chicago) and 95% confidence intervals for all proportions, likelihood ratios and risk ratios were calculated using CIA, Confidence Interval Analysis software. The North Sheffield Research Ethics Committee approved the evaluation of the chest pain unit.

## Results

During the study period 706 patients were assessed on the CPU. Details of the study population characteristics are outlined in table 1. All patients received baseline blood sampling. This was performed at a mean time from symptom onset of 6.2 hours (median 3.0 hours). Repeat blood sampling was performed on 604 patients (86%) at a mean time from symptom onset of 8.6 hours (median 6.5 hours). After CPU assessment 596 (84%) were discharged home and 110 (16%) were admitted. Of those discharged, 515 (86%) returned for review at 72 hours. At review one patient had a previously undetected rise in troponin T (0.8 ng/ml) and was classified as having ACS with myocyte necrosis. All other cases of ACS were detected by the CPU protocol.

**Table 1:** Characteristics of the study population

Mean age	53.6 years (range 22 to 99)
Male	431 (61%)
Known coronary heart disease	150 (21%)
Diabetes	48 (7%)
Hypertension	181 (26%)
Hyperlipidaemia	114 (16%)
Smoker	215 (35%)

ACS with clinical myocardial infarction was ultimately in diagnosed in 30 patients (4.2%), while another 30 (4.2%) had ACS with myocyte necrosis. Over the following six months 32 patients (4.5%) had a major adverse cardiac event. These included eight cardiac deaths, five non-fatal myocardial infarctions, two arrhythmias, and 17 revascularisation procedures.

Serial ECG/ST segment monitoring was performed for 690 patients, baseline CK-MB(mass) was measured in 687, repeat CK-MB(mass) was measured in 601, troponin T was measured in 686 patients, and 422 patients received an exercise treadmill test. Table 2 shows the sensitivity, specificity, positive and negative predictive values and table 3 shows the likelihood ratios of the tests for ACS with clinical myocardial infarction at presentation. The same parameters are outlined for ACS with clinical myocardial infarction or myocyte necrosis in tables 4 and 5. Serial ECG / ST segment monitoring added relatively little to the assessment. None of the positive cases involved ST elevation myocardial infarction requiring reperfusion therapy. All the biochemical markers were useful for ruling in ACS with clinical myocardial infarction and ACS with myocyte necrosis. The second CK-MB(mass) sample and the delta CK-MB(mass) were useful for ruling out myocardial infarction, but did not reliably rule out myocyte necrosis. Using a lower threshold for troponin T positivity markedly improved sensitivity with only a modest loss of specificity. Only troponin T, using a lower threshold, was useful for ruling out ACS with myocyte necrosis. However, it should be recognised that this diagnosis is based upon detection of troponin T.

**Table 2:** Sensitivity, specificity, positive and negative predictive values for the diagnosis of ACS with clinical myocardial infarction at presentation

	Sensitivity (95% CI)	Specificity (95% CI)	Positive predictive value (95% CI)	Negative predictive value (95% CI)
Serial ECG & ST monitoring N = 690	33.3% (19.2 to 51.2)	95.3% (93.4 to 96.7)	24.4% (13.8 to 39.3)	96.9% (95.3 to 98.0)
Initial CK-MB(mass) N = 687	63.3% (45.5 to 78.1)	97.0% (95.3 to 98.0)	48.7% (33.9 to 63.8)	98.3% (97.0 to 99.0)
Delayed CK-MB(mass) N = 601	95.7% (79.0 to 99.2)	96.2% (94.3 to 97.5)	50.0% (35.8 to 64.2)	99.8% (99.0 to 100)
Delta CK-MB(mass) N = 601	95.7% (79.0 to 99.2)	98.4% (97.0 to 99.1)	70.0% (53.4 to 83.9)	99.8% (99.0 to 100)
Any positive CK-MB(mass) N = 601	100% (88.6 to 100)	95.6% (93.7 to 96.9)	50.8% (38.3 to 63.2)	100% (99.4 to 100)
Troponin T > = 0.1 ng/ml N = 686	83.3% (66.4 to 92.7)	98.8% (97.7 to 99.4)	75.8% (59.0 to 87.2)	99.3% (98.3 to 99.7)
Troponin T > = 0.03 ng/ml N = 686	96.4% (82.3 to 99.4)	95.9% (94.1 to 97.2)	50.0% (37.1 to 62.9)	99.8% (99.1 to 100)

**Table 3:** Likelihood ratios for the diagnosis of ACS with clinical myocardial infarction at presentation

	Positive likelihood ratio (95% CI)	Negative likelihood ratio (95% CI)
Serial ECG & ST monitoring N = 690	7.1 (3.8 to 13.1)	0.700 (0.543 to 0.901)
Initial CK-MB(mass) N = 687	20.8 (12.5 to 34.7)	0.378 (0.236 to 0.605)
Delayed CK-MB(mass) N = 601	25.1 (16.5 to 38.2)	0.045 (0.007 to 0.307)
Delta CK-MB(mass) N = 601	61.4 (39.1 to 118.1)	0.044 (0.006 to 0.300)
Any positive CK-MB(mass) N = 601	~25*	~0*
Troponin T > = 0.1 ng/ml N = 686	70.4 (34.1 to 140.8)	0.169 (0.079 to 0.387)
Troponin T > = 0.03 ng/ml N = 686	23.5 (16.1 to 34.2)	0.037 (0.005 to 0.255)

*Unable to calculate precisely as no false negative were recorded

**Table 4:** Sensitivity, specificity, positive and negative predictive values for the diagnosis of ACS with myocyte necrosis or clinical myocardial infarction at presentation

	Sensitivity (95% CI)	Specificity (95% CI)	Positive predictive value (95% CI)	Negative predictive value (95% CI)
Serial ECG & ST monitoring N = 690	23.3% (14.4 to 35.4)	95.7% (93.8 to 97.0)	34.1% (21.6 to 49.5)	92.9% (90.7 to 94.6)
Initial CK-MB(mass) N = 687	43.3% (31.6 to 55.9)	97.9% (96.5 to 98.8)	66.7% (51.0 to 79.4)	94.8% (92.8 to 96.2)
Delayed CK-MB(mass) N = 601	63.5% (49.9 to 75.2)	98.0% (96.4 to 98.9)	75.0% (60.6 to 85.4)	96.6% (94.7 to 97.8)
Delta CK-MB(mass) N = 601	55.8% (42.3 to 68.4)	99.6% (98.7 to 99.9)	93.5% (79.3 to 98.2)	96.0% (94.0 to 97.3)
Any positive CK-MB(mass) N = 601	71.7% (59.2 to 81.5)	97.4% (95.9 to 98.4)	72.9% (60.4 to 82.6)	97.3% (95.7 to 98.3)
Troponin T > = 0.1 ng/ml N = 686	55.0% (42.5 to 66.9)	100% (99.4 to 100)	100% (89.6 to 100)	96.0% (94.2 to 97.2)
Troponin T > = 0.03 ng/ml N = 686	89.7% (79.2 to 95.2)	99.7% (98.8 to 99.9)	96.3% (87.5 to 99.0)	99.6% (97.9 to 99.9)

**Table 5:** Likelihood ratios for the diagnosis of ACS with myocyte necrosis or clinical myocardial infarction at presentation

	Positive likelihood ratio (95% CI)	Negative likelihood ratio (95% CI)
Serial ECG & ST monitoring N = 690	5.4 (3.0 to 9.8)	0.801 (0.696 to 0.922)
Initial CK-MB(mass) N = 687	20.9 (11.3 to 38.5)	0.579 (0.464 to 0.722)
Delayed CK-MB(mass) N = 601	31.7 (17.0 to 58.9)	0.373 (0.261 to 0.534)
Delta CK-MB(mass) N = 601	153.1 (37.6 to 623.5)	0.444 (0.327 to 0.602)
Any positive CK-MB(mass) N = 601	28.1 (16.9 to 46.7)	0.291 (0.194 to 0.435)
Troponin T > = 0.1 ng/ml N = 686	~25*	~0*
Troponin T > = 0.03 ng/ml N = 686	281.5 (70.4 to 1126)	0.104 (0.049 to 0.221)

*Unable to calculate precisely as no false positives were recorded

Table 6 shows the relative risk of all cardiac events over the subsequent six months for each diagnostic test and table 7 shows the relative risk of cardiac death, non-fatal AMI or arrhythmia. Positive results for serial ECG / ST segment monitoring or biochemical cardiac markers showed a weak association with cardiac events over the following six months. This association disappeared when revascularisation procedures were excluded from the definition of cardiac events. Positive exercise stress test result showed a much stronger association with cardiac events, which was maintained even when revascularisation procedures were excluded.

**Table 6:** Relative risk of a cardiac event over the following six months

Test	Event rate: positive test	Event rate: negative test	Relative risk (95% CI)
Serial ECG & ST monitoring N = 690	5/41	27/649	2.72 (1.10 to 6.74)
Initial CK-MB(mass) N = 687	5/39	27/648	2.84 (1.15 to 7.02)
Delayed CK-MB(mass) N = 601	5/44	23/557	2.57 (1.02 to 6.47)
Delta CK-MB(mass) N = 601	3/31	25/570	2.10 (0.67 to 6.61)
Any positive CK-MB(mass) N = 601	8/59	24/628	3.24 (1.52 to 6.93)
Troponin T > = 0.1 ng/ml N = 686	8/33	24/673	6.80 (3.31 to 13.96)
Troponin T > = 0.03 ng/ml N = 686	9/54	23/632	4.58 (2.23 to 9.40)
Exercise treadmill test N = 422	9/37	4/385	19.03 (6.10 to 59.34)

**Table 7:** Relative risk of cardiac death, non-fatal myocardial infarction or arrhythmia over the following six months

Test	Event rate: positive test	Event rate: negative test	Relative risk
Serial ECG & ST monitoring N = 690	1/41	15/649	1.06 (0.14 to 7.79)
Initial CK-MB(mass) N = 687	0/39	16/648	-
Delayed CK-MB(mass) N = 601	1/44	13/557	0.97 (0.13 to 7.28)
Delta CK-MB(mass) N = 601	0/31	14/570	-
Any positive CK-MB(mass) N = 601	1/59	15/628	0.71 (0.09 to 5.28)
Troponin T > = 0.1 ng/ml N = 686	1/33	15/673	1.36 (0.18 to 9.98)
Troponin T > = 0.03 ng/ml N = 686	2/54	14/632	1.67 (0.39 to 7.17)
Exercise treadmill test N = 422	2/37	3/385	6.63 (1.14 to 38.50)

## Discussion

These findings suggest that serial ECG recording and ST segment monitoring, as applied in our protocol, add little diagnostic value in patients with a normal or non-diagnostic initial ECG. Only a minority of cases of ACS were detected by ECG monitoring and most of the positive results from this test were false positives. Serial ECG recording and ST segment monitoring offers the potential advantage of allowing early detection of ST segment elevation myocardial infarction that may be eligible for thrombolysis [6], but no such cases occurred in our cohort. Our findings are consistent with those of Decker et al [7] who found that serial ECG was of limited value in their CPU protocol. It should be appreciated, however, that our protocol used a variable duration of ST segment monitoring and a variable number of serial ECGs, with some patients receiving only two hours of monitoring and two serial ECGs. Hence these findings may not apply to more prolonged regimes, or to populations with a higher prevalence of ACS.

Baseline CK-MB(mass) testing allows early detection of ACS and calculation of a CK-MB(mass) rise. However, the initial CK-MB(mass) alone has insufficient sensitivity to rule out ACS. The addition of a repeat sample at least two hours later and at least six hours after the onset of pain provides adequate sensitivity to rule out ACS with myocardial infarction, but not myocyte necrosis. Our estimate of sensitivity for myocardial infarction is consistent with previously published estimates [3,8,9]. If we simply wish to rule out myocardial infarction then this would appear to be a relatively cheap and effective way of achieving that aim.

Using a traditional threshold for positivity of 0.1 ng/ml for troponin T [10,11] in this protocol does not reliably rule out myocardial infarction or myocyte necrosis. However, if a lower threshold of 0.03 ng/ml is used then 96% of cases of myocardial infarction and almost 90% of cases of myocyte necrosis will be detected. This finding is consistent with previously published data showing that use of a lower threshold for positivity for troponin T is associated with improved early sensitivity [12]. However, since we tested this threshold as a post hoc analysis, further prospective validation of the performance of a lower threshold is required. Furthermore, lowering the threshold would be expected to reduce specificity and lead to more false positives being generated. Potential incorporation bias (see limitations section) means that it is difficult to determine the impact of altering the threshold upon specificity.

The question of whether to use CK-MB(mass) or troponin T or both is a subjective judgement in which the benefits of detecting and treating cases of ACS must be weighed against the costs of additional testing and management of false positives. ACS with myocardial infarction is associated with a markedly increased risk of adverse events[5,10] and thus an increased expectation of benefit from treatment [13]. The risks of ACS with myocyte necrosis are smaller, but still indicate potential benefit from detection and treatment [5]. In estimating the potential costs and benefits of detecting cases of ACS we also need to consider prevalence. CPU patients have a low prevalence of ACS, so a large number of additional patients will need to be tested to detect a small number of additional cases.

The value of exercise treadmill testing in a CPU protocol has been questioned [2]. It may be costly to implement and risks generating large number of false positives due to poor specificity. This study has shown that exercise treadmill testing offers useful prognostic information that is not provided by ECG or biochemical testing. Meanwhile, concerns about false positives are undermined by evidence from a recent randomised controlled trial [14] in which two-thirds of patients randomised to CPU care received an exercise treadmill test compared to only one-third of the routine care group. Despite this difference the rate of referral for angiography was identical. Whether the additional prognostic information provided by treadmill testing justifies the addition cost remains debatable.

This study has a number of limitations that should be appreciated. The reference standard for diagnosis was not independent of the tests being evaluated. Troponin elevation was the diagnostic criterion for ACS so estimates of sensitivity and particularly specificity will be subject to incorporation bias. Any patient who has an elevated troponin will, by definition, have a diagnosis of ACS, unless repeat sampling shortly afterwards is negative. Therefore specificity of troponin, using this definition of ACS, is expected to be high. The value of our analysis of troponin therefore lies in assessment of early sensitivity, particularly in comparing the early sensitivity of using different thresholds for positivity.

Caregivers were aware of the results of all the tests under evaluation and thus would be more likely to rigorously follow-up those with positive tests, raising the possibility of work-up bias. Patients with positive tests were admitted while those with negative tests were discharged. Although 86% of discharged patients attended review at 72 hours, it is possible that some patients with false negative initial tests may have failed to attend follow-up, and were thus misclassified as true negative. A similar bias may influence the association between test results and adverse events when revascularisation procedures are included in the definition, but not the association between treadmill testing and adverse events limited to cardiac death, non-fatal myocardial infarction or arrhythmia.

Statistical analysis inevitably requires some simplification of the data used. For ST segment monitoring / serial ECG and for exercise treadmill testing this involved categorising all results into positive or negative. For exercise treadmill testing inconclusive results were classified as negative. This approach may lead to under-estimation of the diagnostic value of the tests. Furthermore, since patients with new abnormalities on their ECG were excluded from CPU evaluation, it is perhaps not surprising that further ECG-based tests had limited value. This again reinforces the importance of not extrapolating these findings to different populations, such as those with a high prevalence of ACS.

## Conclusion

ST segment monitoring and serial ECG recording, as applied in our protocol, appears to add little diagnostic value in patients with a normal or non-diagnostic initial ECG. CK-MB(mass) measurement allows reliable detection of ACS with myocardial infarction but not myocyte necrosis (defined as a troponin elevation without myocardial infarction). Using a low threshold for positivity for troponin T improves sensitivity of this test for myocardial infarction. Exercise treadmill testing offers additional prognostic information regarding the risk of subsequent adverse cardiac events.

## Competing interests

The author(s) declare that they have no competing interests.

## Authors' contributions

SG conceived the idea, planned the study, analysed the data, and wrote the paper. TL, JA and KA collected the data and helped to write the paper. FM helped conceive the idea and write the paper. All authors contributed to the final draft.

## Pre-publication history

The pre-publication history for this paper can be accessed here:


